# The complete mitochondrial genome of sea slug *phyllidia elegans* bergh, 1869 (nudibranchia, phyllidiidae) from the South China sea

**DOI:** 10.1080/23802359.2022.2124827

**Published:** 2022-09-28

**Authors:** Zhehao Li, Xiaoqi Zeng, Gang Ni

**Affiliations:** a Ministry of Education Key Laboratory of Mariculture, Ocean University of China, Qingdao, China; b Institute of Evolution and Marine Biodiversity, Ocean University of China, Qingdao, China

**Keywords:** Mitochondrial genome, phylogenetic analysis, Nudibranchia, sea slug, *Phyllidia elegans*

## Abstract

In this study, the complete mitochondrial genome (mitogenome) sequence of sea slug, *Phyllidia elegans* Bergh, 1869 (Nudibranchia, Phyllidiidae), was sequenced and characterized. The assembled mitogenome was 14618 bp in length, including 13 protein-coding genes (PCGs), two ribosomal RNA genes, and 22 transfer RNA genes. The overall base composition of *P. elegans* mitogenome is 32.1% for A, 13.5% for C, 15.7% for G, and 38.7% for T. The gene order was identical to other Phyllidiid species. Phylogenetic analysis placed *P. elegans* and *Phyllidia oecllata* in one clade.

Nudibranchs, also known as sea slugs, are shell-less and brightly colored marine gastropod mollusks (Penney et al. 2020). They occur throughout the world’s oceans, and their richness can reflect the diversity and health of coral reefs (Xiang et al. 2017; Undap et al. 2019). *Phyllidia elegans* Bergh, 1869 is a species of Nudibranchia, Phyllidiidae, with major distribution in the tropical Indo-Pacific region (Brunckhorst 1993). It has cream-colored tubercles on the dorsum with black longitudinal stripes on the foot sole (Brunckhorst 1993; Dominguez et al. 2007). In this study, we reported the complete mitochondrial genome (mitogenome) of *P. elegans* and examined its phylogenetic position.

The *P. elegans* specimen was collected from the South China Sea (16°12′9.4″ N, 111°40′43.5″ E) through Scuba diving and deposited in Fisheries College, Ocean University of China (https://scxy.ouc.edu.cn/main.htm, contact person: Zeng Xiaoqi, email: zengxq@ouc.edu.cn) under the voucher number HN-XS2108003. Since *P. elegans* is neither an endangered nor a protected species in China, no specific permissions or licenses are required for collection. Total genomic DNA was extracted from foot tissue using Tsingke DNA extraction Kit. The library was constructed and sequenced by the Illumina HiSeq X Ten platform to obtain paired-end reads (150 bp). Clean data was assembled by NOVOPlasty 4.2 software (Dierckxsens et al. 2017) using cox1 gene fragment of *Phyllidiella pustulosa* as a seed sequence. The mitogenome was annotated by MITOS (Bernt et al. 2013) and tRNAscan-SE server (Lowe and Chan 2016).

The complete mitogenome of *P. elegans* was 14618 bp in length and encoded 37 genes including 13 protein-coding genes (PCGs), two ribosomal RNA (rRNA), and 22 transfer RNA (tRNA). The gene order was identical to other reported mitogenomes of Phyllidiid species as follows (e.g., Xiang et al. 2016; Do et al. 2019): cox1, tRNA-Val, rrnL, tRNA-Leu, tRNA-Ala, tRNA-Pro, nad6, nad5, nad1, tRNA-Tyr, tRNA-Trp, nad4l, cob, tRNA-Asp, tRNA-Phe, cox2, tRNA-Gly, tRNA-His, tRNA-Cys, tRNA-Gln, tRNA-Leu, atp8, tRNA-Asn, atp6, tRNA-Arg, tRNA-Glu, rrnS, tRNA-Met, nad3, tRNA-Ser, tRNA-Ser, nad4, tRNA-Thr, cox3, tRNA-Ile, nad2, and tRNA-Lys. Within these genes, thirteen genes (atp8, atp6, nad3, cox3, rrnS, tRNA-Gln, tRNA-Leu, tRNA-Asn, tRNA-Arg, tRNA-Glu, tRNA-Met, tRNA-Ser, and tRNA-Thr) were located on the light strand, while all the others were located on the heavy strand. The base composition of this mitogenome was A = 32.1%, C = 13.5%, G = 15.7%, and T = 38.7%. Most PCGs (cox1, nad5, nad1, cox2, atp8, atp6, nad3, nad4, cox3, and nad2) started with ATG as the start codon, and three genes (nad6, nad4l, and cob) with ATA codon. All PCGs used the conventional stop codons TAA and TAG except one gene nad2 using an incomplete stop codon T.

The mitogenome of *P. elegans* and 16 related species with available mitogenomes on NCBI were used to infer the phylogenetic position of *P. elegans*. Two species *Pleurobranchaea novaezealandiae* and *Berthellina* sp. in the Pleurobranchia were chosen as outgroups. The sequences of each PCG were individually aligned in MAFFT v.7 (Katoh and Standley 2013) with default settings, and ambiguously aligned regions were eliminated using Gblocks v.0.91b (Talavera and Castresana 2007). ModelFinder (Kalyaanamoorthy et al. 2017) selected the following models as the best-fit substitution model under the Bayesian Information Criterion: TVM + F + I + G4 for atp6, atp8, nad2, nad3, nad4, nad4l, nad5, and nad6; GTR + F + I + G4 for cob, nad1, and cox2; TIM + F + I + G4 for cox1 and cox3. A maximum-likelihood (ML) tree based on the 13 PCGs was constructed using IQ-TREE 1.6.8 (Guindon et al. 2010, Nguyen et al. 2015, Chernomor et al. 2016) with 1000 ultrafast bootstrap replicates (Minh et al. 2013). The phylogenetic result showed that *P. elegans* was clustered with *Phyllidia ocellata* in the Phyllidiidae with maximum support of 100% (Figure 1). The mitogenome of *P. elegans* could be useful in further phylogenetic analysis of Phyllidiidae within Nudibranchia.

**Figure 1.**
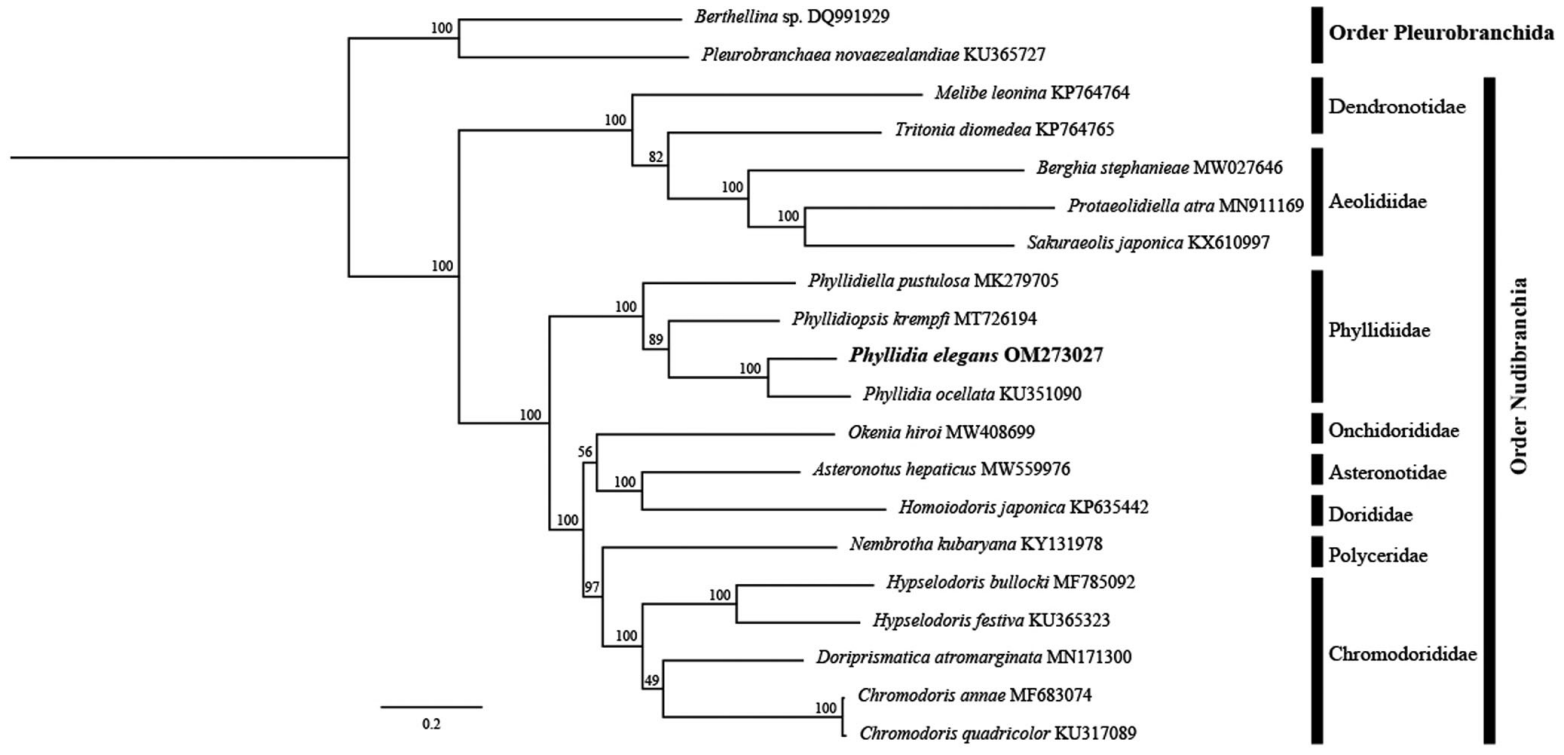
The ML phylogenetic tree based on 13 PCGs of 17 nudibranch species. Two species *Pleurobranchaea novaezealandiae* and *Berthellina* sp. belonging to Pleurobranchia were used as outgroups. Numbers near the nodes represent ML bootstrap value.

## Data Availability

The genome sequence that supports the findings of this study was openly available in GenBank of NCBI under accession No. OM273027 (https://www.ncbi.nlm.nih.gov/search/all/?term=OM273027). BioProject no. PRJNA832552; Biosample: SAMN27914503; SRA no.: SRR18959687.
